# Resistant starch: Implications of dietary inclusion on gut health and growth in pigs: a review

**DOI:** 10.1186/s40104-021-00644-5

**Published:** 2021-11-16

**Authors:** Felina P. Y. Tan, Eduardo Beltranena, Ruurd T. Zijlstra

**Affiliations:** Department of Agricultural, Food and Nutritional Science, University of Alberta, Edmonton, AB T6G 2P5 Canada

**Keywords:** Growth performance, Gut health, Resistant starch, Swine

## Abstract

Starch from cereal grains, pulse grains, and tubers is a major energy substrate in swine rations constituting up to 55% of the diet. In pigs, starch digestion is initiated by salivary and then pancreatic α-amylase, and has as final step the digestion of disaccharides by the brush-border enzymes in the small intestine that produce monosaccharides (glucose) for absorption. Resistant starch (RS) is the proportion of starch that escapes the enzymatic digestion and absorption in the small intestine. The undigested starch reaches the distal small intestine and hindgut for microbial fermentation, which produces short-chain fatty acids (SCFA) for absorption. SCFA in turn, influence microbial ecology and gut health of pigs. These fermentative metabolites exert their benefits on gut health through promoting growth and proliferation of enterocytes, maintenance of intestinal integrity and thus immunity, and modulation of the microbial community in part by suppressing the growth of pathogenic bacteria while selectively enhancing beneficial microbes. Thus, RS has the potential to confer prebiotic effects and may contribute to the improvement of intestinal health in pigs during the post-weaning period. Despite these benefits to the well-being of pigs, RS has a contradictory effect due to lower energetic efficiency of fermented vs. digested starch absorption products. The varying amount and type of RS interact differently with the digestion process along the gastrointestinal tract affecting its energy efficiency and host physiological responses including feed intake, energy metabolism, and feed efficiency. Results of research indicate that the use of RS as prebiotic may improve gut health and thereby, reduce the incidence of post-weaning diarrhea (PWD) and associated mortality. This review summarizes our current knowledge on the effects of RS on microbial ecology, gut health and growth performance in pigs.

## Background

Nursery pig mortality is a complex interplay that involves the animal, and the environment, including diet and infectious etiologies [1]. In the United States, mortality rate of nursery pigs is 3.6% with enteric-related factors (failure to thrive, 22.1% and scours, 9.4%) accounting for nearly one-third of nursery pig deaths (USDA, 2015). Post-weaning diarrhea is a multifactorial disease that occurs within 2 weeks after weaning and is most often characterized by diarrhea, dehydration, reduced growth performance, and mortality [2, 3]. Although PWD can result in mortality of up to 25%, the magnitude of mortality is often mild to moderate ranging from 1.5% to 2% [1, 2]. During the immediate post-weaning period, various factors reducing feed intake have been identified and include dietary, microbiological, environmental factors and their interactions [4]. Refusal to eat and thus reduction in feed intake soon after weaning leads to morphological and functional changes in the intestine resulting in incomplete gut function, subsequent decrease in brush border enzyme activity, and thus absorptive capacity [4–6]. The various stressors and low feed intake compromise gut mucosal integrity causing increased intestinal pathogen permeability, translocation of bacteria, and subsequently inflammation [7]. These compromised digestive and absorptive functions contribute together with inflammation to PWD, dehydration and poor performance [8]. Dietary antibiotic growth promoters and therapeutic doses of zinc oxide and copper sulfate have been used over the last decades to prevent pathogenic diarrhea, maintain health and thus sustain growth performance of pigs. However, in recent years, concerns about antimicrobial resistance and the environmental impact of zinc oxide have prompted the swine industry to explore alternatives [9, 10]. Adopting good animal husbandry practices including nutrition, feeding, management of the nursery environment, biosecurity, and disease prevention are indeed possible strategies to maintain gut health and pig performance without inclusion of dietary antibiotic growth promoters [11]. Diet formulation aspects to consider for optimum gut health include macronutrients (starch, protein, fiber, and fat) and minerals, antioxidant supplementation, and feed additives to modulate host immunity [8]. Specific dietary interventions such as reduction of protein content [12], inclusion of fermentable carbohydrates [13, 14] fiber, and resistant starch (RS) [15] can assist in the weaning transition from liquid milk to dry food. Specifically, RS may be regarded as prebiotic because most forms of RS have features such as stimulating beneficial gut bacteria, increasing total short-chain fatty acid (SCFA), more specifically butyrate levels that confer benefits to the host [16]. However to date, few studies in pigs have examined the efficacy of dietary RS on gut health in comparison with dietary antibiotics or in combination with prebiotics and probiotics [17]. The classification of RS and application of feed processing to alter the value of RS have been discussed in previous reviews [18–21]. The present review addresses the current state of knowledge on the effects of RS on gut health and resulting growth performance in pigs. Nursery and growing pig studies are the focus; some *in* *vitro* studies or *in* *vivo* studies using other monogastric animals (poultry and mice) as animal models are also cited.

## Resistant starch as prebiotic

### Starch chemistry

Starch, the main carbohydrate in cereal and pulse grains, is stored in the amyloplast as discrete granules with distinct morphology in various botanical origins [22]. Starch consists primarily of α-glucans in the form of amylose and amylopectin. Amylose is considered a linear polysaccharide composed of α-D-glucose units with α-1,4-glycosidic linkages with less than 0.5% α-1,6-branching points [23, 24]. Amylopectin is a larger, more branched molecule comprised of α-D-glucose units joined by α-1,4-glycosidic bonds and 5% to 6% α-1,6-glycosidic bonds [25, 26]. Native starches from various botanical origins consist of 18% to 36% amylose and 72% to 82% amylopectin [25, 27, 28]. Starch is deposited as discrete granules in densely packed, concentric layers of growth rings containing alternating crystalline and amorphous regions [24]. Starch granules from a wide variety of plant sources display distinct crystalline structure and susceptibility to enzymatic and chemical reactions [22, 29, 30]. The crystallinity, granule size and surface area, ratio of amylose to amylopectin, porosity, structural inhomogeneities, and degree of integrity influence the susceptibility of the granules to the enzymatic action of α-amylase [31–33]. Waxy starch granules, that are mostly amylopectin, are more readily hydrolyzed compared with granules with a greater content of amylose [34].

### Types of resistant starch

The term resistant starch (RS) was first used to describe the portion of starch that was cooled in cooked foods but was resistant to digestion by α-amylase [35]. This definition was then extended to include starch and its degradation subunits that reach the large intestine and there become substrate for microbial fermentation [36]. Resistant starch was later described as an analogous carbohydrate that should be considered as dietary fiber [27]. Resistant starch is classified based on the source, and physicochemical characteristics of starches [37]. The RS type 1 (RS1) are physically inaccessible starches in pulse grains and cereal grains (Table 1). Amylolytic enzymes are not able to reach these starches located within intact plant cell walls and unprocessed whole grains. The RS type 2 (RS2) are native resistant starch granules found in green banana and raw potato that are easily gelatinized with the presence of water at 60°C [37]. A second type of RS 2 (i.e. high amylose cornstarch) is characterized by its high resistance to gelatinization at temperatures above 120°C required for gelatinization [58, 59]. The RS type 3 (RS3) are retrograded starches that occur when starchy food such as potato and bread are first cooked (gelatinized) and then recoil when cooled. The RS type 4 (RS4) are chemically modified starches that interfere with the action of amylases. For the latter, introduction of chemical bonds occurs through processes that include dextrinization, etherification, esterification, oxidation, and cross-linking utilizing chemical reagents [58, 59]. The RS type 5 (RS5) is mainly associated to amylose-lipid V-type complexes such as starch-fatty acids and starch-monoglycerides that have reduced starch digestibility [60–62].

**Table 1:** Types of resistant starch (RS) and starch sources used in pig studies

Type of RS	Description of RS	Starch sources used in pig studies	References
RS1	Native starch granules entrapped or inaccessible to enzymes	Hull-less barley, high moisture corn, low amylose barley and corn, oat, rice, sorghum, triticale, wheat	[38]
		Field pea	[39]
RS2	Native starch granules with conformation or structure that resist enzymes	Raw potato starch	[15, 40–44]
		Sweet potato	[41]
		Hull-less barley, high moisture corn, low amylose barley and corn, oat, rice, sorghum, triticale, wheat	[38]
		Field pea	[39]
		High-amylose corn starch	[45–53]
RS3	Retrograded starch that occurs naturally during food processing	Tapioca starch	[54, 55]
		Barley	[38, 39]
		Corn	[38]
		Field pea	[39]
		Rice	[38]
		Wheat	[38]
RS4	Chemically-modified starch	Enzymatically-modified starch	[56, 57]

### Starch digestion vs. starch fermentation

Starch digestion begins in the mouth and is initiated by salivary α-amylase during chewing. Approximately 5% of starch is broken down by oral α-amylase due to the short span of time in the mouth [63]. The acidic environment in the stomach restricts starch digestion to a minimum. The luminal phase of starch digestion continues in the small intestine with the secretion of pancreatic α-amylase producing maltose, maltotriose, short-branched oligosaccharides, and α-limit dextrin. Further hydrolysis of disaccharides and oligosaccharides into monosaccharides by the brush border enzymes (maltase-glucoamylase and sucrase-isomaltase) occurs prior to absorption across the small intestinal epithelium [63, 64]. The final digestion product glucose is actively transported across the apical membrane of enterocytes via the ﻿Na^+^/glucose cotransporter 1 against an electrochemical gradient [65]. Less than 10% of glucose is absorbed passively via the paracellular route [66]. The limited studies found in pigs indicate that factors such as weaning age, feed intake, stressors, and diet composition may decrease passive absorption [67]. Glucose then exits the enterocytes at the basolateral membrane via a bidirectional, Na^+^-independent monosaccharide transporter, glucose transporter 2. The Na^+^/glucose cotransporter 1 is the major route for the transport of glucose from the lumen into enterocytes. However, based on studies in rats and cell lines, increasing evidence indicates that glucose transporter 2 can be rapidly recruited to the brush-border membrane when the luminal glucose concentration is high and thereby, increases the capacity of glucose uptake by the enterocytes [68–71]. Starch digestion is influenced by the ratio amylose: amylopectin and branch chain-length distribution of amylopectin [59]. Indeed, starch with high amylose content reduced the rate of starch digestion and thus lowered postprandial blood glucose compared with starch with lower amylose content [49]. The crystalline structure and amylopectin chain length in starches of different botanical origin strongly influenced the rate of starch digestion in an *in vitro* model mimicking porcine digestion [52].

A proportion of starch resists enzymatic degradation in the small intestine and so passes into the large intestine (resistant starch) to be fermented by the resident microbiota to produce SCFA, CO_2_, H_2_, and CH_4_ [72]. The SCFA produced are rapidly absorbed from the lumen either by passive diffusion or active transport utilizing monocarboxylate transporter 1 (MCT1) or Na^+^-dependent monocarboxylate transporter 1 [73]. Oxidation of SCFA contributes approximately 60% to 70% of energy to colonocytes with butyrate as the main energy source, whereas the remaining SCFA are transported across the basolateral membrane by MCT4 [73]. Lesser known MCT isoforms (MCT2 and MCT4) are found in the small intestine and colon of the pig [74].

The most commonly used techniques to assess digestion of starch in pigs are ileal cannulation, portal-vein catheterization, and slaughter techniques [75]. In ileal-cannulated pigs, the insertion of a cannula at the terminal ileum allows for the collection of digesta and feces. Apart from measurements of nutrient digestibility, bacteria and metabolites can be quantified from these samples [76]. The use of indigestible markers such as chromic oxide, allows for the quantification of the extent of starch digestion in the small vs. large intestine. In portal-vein catheterization, pigs are surgically fitted with indwelling catheters in the portal vein and carotid artery, and a flow probe is installed around the portal vein [77]. With this technique, the kinetics of starch digestion and absorption of metabolites such as glucose, lactic acid, short-chain fatty acids, and amino acids can be studied [75]. Alternatively, p-aminohippuric acid can be administered into the bloodstream to measure portal blood flow and nutrient fluxes [78]. The slaughter technique involves euthanizing pigs at a specific time point after meal and followed by prompt collection of digesta samples and sometimes tissue at multiple sites of the gastrointestinal tract [79]. This technique provides a localized progression of starch digestion without surgery, thereby, minimizing the risk of altering gut physiology [75] but is a static portrait of digestion at sampling sites rather than a reflection of the dynamics of the digestion process over time as influenced by peristalsis and nutrient flow.

### Interaction with other feed components

In cereal and pulse grains, several non-starch components that are associated with the starch granules may restrict the digestibility of starch. *In* *vitro* studies have indicated that amylose-lipid complexes reduced the accessibility of α-amylase to amylose for digestion [80]. The rate of α-amylolysis is also affected by the protein-starch structural network that forms in the seed endosperm, many of which are hydrophobic [81, 82]. Starch and phenolic compounds such as tannins, phenolic acids, flavonoids, and lignans also interact through hydrophobic and hydrogen bonds to inhibit gastrointestinal enzyme activity to different extents depending on the type of phenolic compounds and type of starch [83, 84]. Non-starch polysaccharides such as mixed-linked β-glucans present in some cereals such as barley and oat grain encapsulate both protein and starch, thereby, decreasing enzyme accessibility, reducing starch digestion and the rate of postprandial glycemia [85, 86].

## Effect of resistant starch on gut health

### Gut microbial profile and diversity

In pigs, the estimated size of the microbial population is approximately 10^10^ to 10^11^ per gram of gut content [87–89]. The largest proportion of bacteria in the pig intestinal microbiome is from the phyla Firmicutes, followed in descending order by Bacteroidetes, Proteobacteria, Actinobacteria, and Spirochaetes [90, 91]. Firmicutes and Bacteroidetes constitute 90% of the microbiota with *Prevotella* from Bacteroidetes phyla being the predominant genus [91, 92]. In the ileal digesta of pigs, the major phylas are Firmicutes and Proteobacteria, with facultative anaerobes *Lactobacillus* and Enterobacteriaceae being the most dominant [92, 93]. The phyla Firmicutes, Bacteroidetes, and Proteobacteria comprise together 90% of the microbiota in pigs at 7-day post-weaning and 84% at 27-day post-weaning. In both post-weaning age groups, *Lactobacillus* was the predominant genus (46%-58%), followed by *Prevotella* (16%-30%) in both the jejunum and colon [92]. As post-weaning age increases, the relative abundance of Proteobacteria phyla decreases. This phylum consists of many pathogenic, gram negative bacteria such as *Escherichia, Salmonella, Vibrio, Helicobacter, and Campylobacter*, all of which are often negatively associated with gut health [90, 91]. Enterotoxigenic *E. coli* proliferation is widely known as a main cause of PWD [94]. Recent findings of high abundance of *Campylobacter* in 7-day post-weaning piglets with diarrhea indicates that these bacteria may also play a relevant role in PWD [92].

**Fig. 1:**
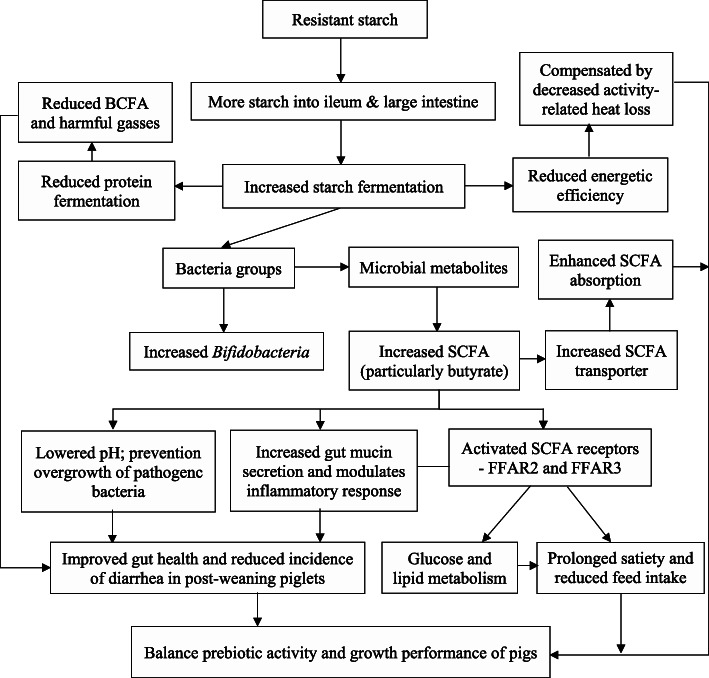
Schematic illustration of the effects of resistant starch on gut health and growth in pigs

The composition and activity of microbes is largely influenced by diet, especially dietary carbohydrates [95–97]. Genes coding for enzymes involved in starch, β-glucan, xylose, and arabinose digestion were particularly enriched in fecal samples from weaned pigs [97]. Metagenomic analyses revealed that Firmicutes possess the extracellular α-(1,4)-glucan branching enzyme and Bacteroidetes possess the periplasmic neopullulanase and α-glucosidase enzymes for starch fermentation in the hindgut [98]. Diets containing purified native starches with amylose ranging from 0 to 80% of starch did not affect the microbial diversity in the large intestine of grower pigs; however, high amylose corn starch (80%) selectively promoted the incidence of *Bifidobacterium* spp. in feces (Table 2) [93]. In weaned pigs, increasing amylose from 0 to 63%, increased *Bifidobacterium* spp. but decreased *Clostridia* clusters IV and XIVa in cecal and colonic digesta [51]. Previously, high amylose corn starch (85%) increased lactobacilli population in the hindgut of weaned pigs [47]. A meta-analysis of 24 research articles indicated that increasing RS type 2 starch with a minimal dietary content of 10% of this in pigs, reduced pH and promoted lactobacilli and bifidobacteria in the feces; thus, potentially limiting the growth of pathogenic bacteria in the hindgut [99] (Fig. 1). In one study, feeding weaned pigs 14% of raw potato starch (RPS) reduced the richness and diversity of the microbial species in the colon [15]. Consumption of a RS type 3 starch diet lowered Firmicutes:*Bacteroidetes* ratio in digesta of the proximal colon in pigs, increased abundance of butyrate-producing *Faecalibacterium prausnitzii*, and decreased pathogenic members of Gammaproteobacteria such as *Escherichia coli* [54]. In pigs, differences observed in cecal and colonic microbiota are likely due to the alteration in the chemical structure of resistant starch at different intestinal sites [54]. The changes in microbial profile with the alteration of dietary starch content and structure supports the concept that the amount and rate of starch that is available for fermentation in the distal region of the gut selectively increased the beneficial bacteria in the gut [101]. Finally, evidence was provided recently for a link between porcine gut microbiota and growth and feed efficiency. Certain bacterial taxa (e.g., *Treponema*, *Methanobrevibacter*, and *Lactobacillus*) that are involved in nutrition digestion, energy harvest, and anti-inflammatory effects are indeed consistently associated with improvements in productivity [102].

**Table 2:** Microbial effects of resistant starch (RS) in pig studies

Test animals^a^	Types of RS	Microbial effects^b^	References
Nursery pigs	RPS (RS 14%)	Increased colonic lactobacilli and *Bacteroides*	[15]
Nursery pigs	Purified corn starch (63% amylose)	Increased cecal and colonic *Bifidobacterium* Decreased *Clostridium* clusters IV and XIVa	[51]
Nursery pigs	High amylose corn starch (85% amylose)	Increased proximal colonic Lactobacilli and Bifidobacteria	[47]
Growing pigs	RS2 in corn, potato, barley, pea, tapioca	Increased fecal lactobacilli and bifidobacteria (meta-analysis)	[99]
Growing pigs	High amylose corn and RPS (RS 11%)	No significant changes in microbial composition	[42]
Growing pigs	Retrograded tapioca starch (RS 34%)	Increased colonic *Ruminococcus bromii*, and bacterial group *Clostridium* cluster IV, IX, XV, XVI, and XVII Decreased colonic Gammaproteobacteria	[54]
Growing pigs	Retrograded tapioca starch (RS 33%)	Increased Lachnospiraceae- and *Ruminococcus*-affiliated phylotypes	[55]
Cannulated growing pigs	Purified corn starch (63% amylose)	Increased fecal *Bifidobacterium*	[93]
Growing-finishing pigs	Purified RPS (RS 13 to 15%)	Increased *Coprococcus*, *Ruminococcus*, and *Turicibacter* Decreased *Sarcina* and *Clostridium*	[43]
Gestating and lactating sows	Pea starch (RS 5 to 9%)	Increased *Bifidobacterium* and ratio of Firmicutes to Bacteroidetes	[100]
Pregnant sows	RPS (RS 5%)	Increased fecal *Clostridia*	[82]

^a^BW of pigs at the start of the study: nursery pigs, 6 to 27 kg; growing pigs, 30 to 63 kg; finishing pigs, 70 kg and above.

^b^Microbial composition represented by relative abundance.

### Gut microbial mediators

Plant cell wall polysaccharides, oligosaccharides, and resistant starches that are not digested by endogenous enzymes in the small intestine are degraded by microbial polysaccharidases and glycosidases to monomers such as glucose, which are then fermented to produce SCFA (mainly acetate, propionate, and butyrate) and gases CO_2_, H_2_, and CH_4_ [103]. Acetate is mainly taken up by the liver to be used as an energy source and as precursor for the synthesis of fatty acids and cholesterol. Propionate is used mainly as a precursor for gluconeogenesis in the liver, kidney, muscle and intestine. Butyrate is the preferred energy source for colonocytes, whereas the remainder SCFA are oxidized by hepatocytes [104, 105]. Minor fermentation products such as ethanol, lactate, and succinate are intermediate products that are metabolized through cross-feeding interactions between bacterial species in the large intestine [106].

The greatest microbial activity occurs in the cecum and proximal colon of the pigs; however, substantial microbial activity also takes place in the distal section of the small intestine [42, 107, 108]. The amount and type of substrate (typically fermentable carbohydrate) affects the microbiota population, thereby, the type and amount of SCFA produced [73, 103, 109]. Actual intestinal SCFA production, however, is not reflected by SCFA concentration in the gut because SCFA are rapidly absorbed (> 95%) from the gut lumen and metabolized by the host [105, 107].

Across pig studies, diets high in RS or amylose increased intestinal SCFA concentrations. Slowly digestible starch (63% amylose) increased ileal starch and total butyrate content [49], and SCFA concentration in the cecum and feces [51]. Both RS type 2 and RS type 3 starch also increased cecal and fecal SCFA [44, 54]. The increase in butyrate production has been positively associated to the increased in SCFA transporter, MCT1 mRNA abundance in some pig studies [54, 110].

At weaning, refusal to eat or drastic reduction in feed intake decreased villus height and increased crypt depth, thus reducing brush-border enzyme activities and nutrient digestion [111, 112]. Other than carbohydrates, undigested protein is also expected to reach the hindgut to a greater extent during the post-weaning period. This undigested protein, along with protein of endogenous origin, is available for fermentation by bacteria resulting in greater protein catabolic activity, and increased formation of SCFA, branched-chain fatty acids (mainly isobutyrate, isovalerate, and 2-methylbutyrate), and gasses such as ammonia, phenols, indoles, and amines that are potentially harmful [109] (Fig. 1). The major amino acid-fermenting bacteria in the gut of pigs are members of Proteobacteria *(E. coli, Klebsiella* spp*.),* Firmicutes *(Streptococcus spp., Clostridium bifermentans, Clostridium difficile, Clostridium perfringens, Megasphaera elsdenii,* and *Acidaminococcus fermentans),* Bacteroidetes [113]. Protein fermentation is not limited to only the hindgut but may happen to a lesser extent in the distal small intestine. Some of the bacteria that have been specifically identified in the small intestine of pigs include *Escherichia coli*, *Klebsiella* spp., *Streptococcus* spp., *Megasphaera* spp., and *Acidaminococcus fermentans* [114]. *Akkermansia muciniphila*, a mucin-degrading bacteria, contributed substantially to nitrogen cycling in the small intestine [115]. Previous studies have indicated that synthesis of bacterial protein primarily occurs in the distal small intestine whereas bacterial protein catabolism mostly occurs in the large intestine. This rate of bacterial protein synthesis and degradations seems to depend on the availability of fermentable carbohydrates [114, 116, 117]. Increasing dietary fermentable carbohydrates shifted nitrogen excretion from urine to feces, thus reducing potentially harmful protein metabolites such as ammonia [118].

### Gut pH

The interaction between diet, intestinal environment and gut bacteria is complex: fermentation of RS leads to increased production of SCFA, which lowers gut pH, thereby affecting microbial composition and in turn, affects both the type and production rate of SCFA [73] (Fig. 1). High amylose starch increased intraluminal SCFA concentrations and lowers pH in digesta and feces of pigs [47, 51]. Greater SCFA concentration from ileum to cecum lowers this gut section pH and therefore prevents overgrowth of pathogenic Enterobacteriaceae and *Clostridia* [119, 120]. As fermentable substrates progressively deplete in the distal part of the large intestine, SCFA concentration declines along from the cecum to the distal colon, and pH increased from 5.6 to 6.6 [121]. The increased pH caused a microbial shift from butyrate-producing bacteria such as *Roseburia* spp. and *Faecalibacterium prausnitzii* to acetate- and propionate-producing bacteria (mainly Bacteroides) [122, 123].

### Gut immune response

In pigs, important gastrointestinal development occurs in the first three months after birth [124, 125]. The establishment of the epithelial barrier coincides with the development of the enteric nervous system and maturation of the adaptive and innate immune system [124, 125]. In commercial pig production, pigs are generally weaned between 14 to 30 days. Other stressors associated with weaning (such as maternal separation, establishment of social hierarchy, disease, and environmental challenges) occurs during the critical period of gastrointestinal barrier development, thereby, affecting the immune maturation processes in the intestinal tract [124]. The effects of weaning on gastrointestinal health are well documented; however, the underlying mechanisms are not fully understood. Weaning stress in pigs has been proposed to trigger corticotropin-releasing factor leading to mast cells activation and release of mast cell mediators such as proteases and tumor necrosis factor (TNF-α) [124]. Initiation of this process in turn reduces tight junctions and increases intestinal permeability allowing the translocation of bacteria and microbial products [124–126]. Several animal studies demonstrated that stress due to weaning induced impaired intestinal barrier function, reduced mRNA expression of tight junction proteins (occludin, claudin-1, and zonula occludens-1) and upregulated expression of pro-inflammatory cytokines (TNF-α and interleukin (IL)-6; Table 3).

**Table 3:** Immunomodulatory effects of resistant starch (RS) in animal studies^a^

Test animal	Type of RS	Immune effects	References
Grower pigs	High amylose corn and RPS (RS 11%)	Increased TNF-α, MCP2, MUC2	[110]
Grower pigs	Retrograded tapioca starch (RS 34%)	Increased PPARγ Decreased NFKB, TLR4, BCL6, ICOS, and CR2	[127]
Grower pigs	RPS (RS > 13%)	Increased IL-1β, F7, PolIII, TLR6 Decreased CD4, ITGB3, CTSB, C1S, SERPING1, TLR7	[43]
Grower pigs	RPS (RS 15.3%)	Increased *MUC4*, *MUC5AC*, and *MUC12*	[128]
Finishing pigs	High amylose corn starch (68.5% amylose)	Increased IL-6, IL-12, IL-1β, and TNF-α in pigs with *Prevotella*-rich enterotype	[129]
Pregnant sows	Pea starch (Gestation RS 5.4%; Lactation RS 8.6%)	Increased zonula occludens-1 in piglets	[100]
Pregnant sows	RPS (RS 5 %)	Increased MUC2, IL6, DEF1B, cecal immunoglobulin A, and regulatory T cells	[44]
Ducks	RPS (RS 3.8%, 7.4% and 19.5%)	Increased claudin-1, zonula occludens-1, MUC2, reduced plasma TNF-α, IL-1β and endotoxin (RS 7.4%)	[130]

^a^*BCL6* B-cell lymphoma 6, *CD4* Cluster of differentiation 4, *CR2* Complement receptor type 2, *CTSB* Cathepsin B, *C1S* Complement component 1S, *DEF1B* Defensin beta 1, *ICOS* Inducible T cell co-stimulator, *IL* Interleukin, *ITGB3* Integrin subunit beta 3, *MCP2* Monocyte chemoattractant protein 2, *MUC* Mucin, *NFKB* Nuclear factor kappa B, *PolIII* RNA polymerase III, *PPARγ* Peroxisome proliferator-activated receptor, *RPS* Raw potato starch, *SERPING1* Serpin Family G Member 1, *TLR* Toll like receptor, *TNF-α* Tumor necrosis factor alpha

In addition to the potential to alter the diversity and stability of gut microbiota, RS affects the intestinal expression of genes involved in immune regulation and thereby gut health. Among SCFA, especially butyrate produced by RS fermentation serves as the primary energy source for the colonocytes, and regarded as the modulator of intestinal barrier function and immunity [131, 132]. Recently, finishing pigs with *Prevotella*-rich enterotype that were fed a diet containing high amylose (68% amylose) corn starch had decreased gene expression of inflammatory cytokines in the colonic mucosa [129]. Feeding a diet containing 34% RS suppressed genes (Table 3) that are involved in many of the immune response pathways in adaptive and innate immune system [113]. In multiple studies, mainly in cell lines and mice, the potential of SCFA in regulating T-cell metabolism and controlling gut inflammatory responses was established [133–137]. Butyrate suppressed pro-inflammatory cytokine production, therefore, suppression of immune response in a high RS diet is likely due to increased SCFA concentration [138, 139]. Long-term intake of RS may affect the immune response. Pigs fed sequentially a diet containing 23% RPS during the growing phase and 28% RPS during the finishing phase, increased expression of colonic mucin genes (*MUC4*, *MUC5AC*, and *MUC12*) compared with feeding a corn starch diet. Therefore, long-term intake of RS may consequentially improve gut health by increasing mucin secretion and lowering the incidence of bacterial products translocation across the gut barrier [128, 140]. Using transcriptomic analysis, pigs that were fed a diet containing 13.3%-15.4% of RS for 100 days altered colonic expression of genes involved in immune response; however, expression of the pro-inflammatory cytokine gene IL-1β also increased. Hence, long-term intake of RS may exert both positive and negative effects on gut health [43]. Pro-inflammatory cytokine signaling has also been linked to increased protein fermentation and down-regulation of MCT1 in the colon of pigs [141].

## Effect of resistant starch on energy metabolism and growth performance

### Metabolic responses

Apparent total tract digestibility of starch is mostly complete; however, apparent ileal digestibility of starch differs depending on its botanical origin [142, 143]. Feeding pigs a slowly digestible starch such as yellow field pea starch (67% inclusion) and RPS (32.5% to 65%) decreased net portal flux of glucose but simultaneously increased net portal flux of SCFA [144, 145]. As established, diets high in RS (high amylose) can increase substrate flow into the hindgut thereby promoting microbial fermentation evidenced by increased total SCFA concentration [47, 51, 93]. Pigs fed diets containing high amylose corn starch [93], raw potato starch [145], and slowly degradable yellow field pea starch [144] have decreased glucose absorption. These findings indicate that in pigs fed a diet containing slowly digestible purified starch of corn, raw potato, or field pea, the intestinal wall metabolizes more glucose, or a portion of dietary starch is fermented in the small intestine. Thus, digestibility of starch alone does not explain the rate of glucose absorption. Previously, *in vitro* starch digestion was used to accurately predict the kinetics of net portal vein appearance of glucose in pigs by applying the corrections for gastric emptying and intestinal glucose utilization [146].

Resistant starches affect host glucose and lipid metabolism by altering the profile of metabolites in systemic circulation such as glucose, cholesterols, triglycerides, and SCFA. Pigs fed diets containing a wide range of amylose or RS have a reduction in postprandial glucose and insulin responses, hence, indicating that a specific starch characteristic may not entirely explain the starch effect on postprandial glucose. In portal vein-catheterized pigs, the net portal appearance of glucose, insulin, C-peptide, and glucose-dependent insulinotropic polypeptide was lowered in pigs fed slowly digestible starch [93]. Insulin secretion peaked prior to peak glucose absorption when pigs were fed a rapidly digestible starch. The presence of glucose in the intestinal lumen facilitates incretin secretion, which accounts for 50% to 70% of the insulin secreted [147]. Two incretins hormones that are important for metabolism of starch metabolites are a) glucose-dependent insulinotropic polypeptide that is secreted from K cells predominantly located in the duodenum, and b) proximal jejunum and glucagon-like peptide-1 (GLP-1) secreted from L cells at the distal small intestine and colon [148, 149]. The other likely reason is the presence of intestinal glucose sensor(s), taste receptor type 1 family (T1R2 + T1R3) in the lumen of the intestine in the presence of free digesta glucose [150, 151].

In mammalian species, evidence is accumulating that SCFA production directly affects pancreatic secretion of insulin [152]. The SCFA may act as signaling molecules by activating its receptors, G-protein coupled receptors 43 (FFAR2), and G-protein coupled receptors 41 (FFAR3) that triggers cell-specific signaling cascades [153]. These receptors are expressed in the ileum, colon, and insulin-sensitive tissues such as adipose tissue, skeletal muscle, liver, and pancreas, thereby, the potential involvement in regulation of glucose and lipid metabolism [73]. Activation of FFAR2 and FFAR3 stimulates the secretion of the incretins GLP-1 and peptide YY (PYY) [154–156]. The PYY, a satiety-related hormone produced by the L-cells in the colon, reinforces the action of insulin by increasing glycemic control in rats [157–159]. In rats, increased FFAR2 was associated with increased GLP-1 in the proximal colon [160]. Furthermore, butyrate had a slower potency than acetate and propionate in stimulating GLP-1 secretion in the basolateral membrane of the colonic cells [161]. In pigs fed a diet with high RS (34%), expression of FFAR2 and FFAR3 did not differ; however, the possibility that receptors were activated is not excluded [162]. Production of SCFA does not seem related to expression of proglucagon, but increased net portal SCFA from fermentation of RS increasing net portal GLP-1 and decreasing glucose-dependent insulinotropic polypeptide in pigs [49].

An earlier study in humans identified that dietary inclusion of 5.4% RS (high amylose corn starch) increased fermentation products, SCFA, and fat oxidation, and thereby may decrease fat accumulation in the long term [163]. In pigs, glucose and insulin are potent signals that upregulates the expression of lipogenic enzymes [164, 165]. In weaned pigs, starch with faster digestion rate (68.9% total starch; 3.5% amylose and 96.5% amylopectin) produced more postprandial blood glucose, insulin, and circulating lipids (triglyceride, total cholesterol, low-density lipoprotein cholesterol, and high density lipoprotein cholesterol) [166]. In addition, the expression of primary lipogenic enzymes, fatty acid synthase, acetyl CoA-carboxylase, and ATP-citrate lyase in the liver and adipose tissue, were increased in pig fed highly digestible starch [166]. Indeed, weaned pigs fed cassava starch containing 80% amylopectin had a greater insulinemic response than pigs fed corn starch containing 70% amylopectin and stimulated lipogenesis in the liver [167]. Recently, oral administration of SCFA to growing pigs prevented fat deposition [168]. More specifically, acetate downregulated expression of genes involved in de novo synthesis of fatty acids; fatty acid synthase, acetyl CoA-carboxylase, and ﻿sterol regulatory element binding protein 1c (SREBP-1C), whereas butyrate enhanced expression of genes involved in fatty acid oxidation; hormone-sensitive lipase and carnitine palmitoyltransferase 1. In finishing pigs, pea starch with high amylose content (51.1% total starch; 28% amylose and 72% amylopectin) consistently induced downregulation of lipogenesis [169].

### Whole body energy metabolism

Previously, RS that is fermented to SCFA was shown to be approximately 17% less efficient than starch that are digested and absorbed as glucose from the small intestine [170]. In the net energy system, energy efficiency from fermented starch is 70% compared with *in-vitro* enzymatically-degradable starch [171]. Using indirect calorimetry, the net energy value of fermented RS (retrograded corn starch) was 83% of the net energy value of enzymatically degradable starch [172]. The differences in energetic efficiency raises the question if switching from digested starch to fermentable starch reduces growth and feed efficiency. Most starch sources (both RS and digested starch) are almost completely degraded in the gastrointestinal tract reaching 100% apparent total tract digestibility; however, apparent ileal digestibility of these starch sources varies: cereal grains (84.8% to 99.4%), pulse grains (78% to 91%), purified starches (69.7% to 97.5%), and tuber (78% to 91%) [173]. The equation that predicts net energy value recommended in North America utilizes total starch as analyzed without considering its actual energetic efficiency [174, 175]. However using total analyzed starch in the equation is a major limitation because, as per the foregoing discussion, starch digestion and starch fermentation cannot be physiologically unlinked. Indeed, differences in site, extent or kinetics of starch digestion affect the energetic efficiency of digested vs. fermented starch due to changes in absorption and subsequent metabolism that affect energy loss through heat production [49, 147, 173]. Part of the lower energetic efficiency of RS due to increased heat increment may be compensated by reducing energy losses due to activity-related heat production that varies between 5% to 35% of maintenance heat production [176–179] (Fig. 1).

### Feed intake and growth

Feed intake regulation likely involves two processes: (1) rate of passage of digesta or transit time [180] and (2) activation of the brain satiety and hunger center as a result of feed consumption, digestion, and metabolites production [180, 181]. The rate of macronutrient digestion that determines the site of digestion and also feed intake control through modification of satiety provide opportunities to improve feed efficiency [182]. Currently, some evidence exists that RS can decrease appetite and short-term food intake; however, the underlying mechanisms are not clearly understood, and findings have been inconsistent. Potential mechanisms include first, SCFA produced by hindgut fermentation stabilize postprandial glucose preventing a decline below basal glucose levels thereby prolonging satiety [183]. Second, luminary SCFA stimulate the release of incretins GLP-1 and PYY that promote satiety via a neural inhibitory effect in the brain [73, 182, 184]. Slow digestion of starch towards the end of the small intestine activates an ileal physicochemical brake switch causing delayed gastric emptying and thus, stimulating the satiety center in the brain via hormonal and neural signals [185]. The SCFA may also activate FFAR2 to release serotonin in the colon, thereby contributing further to satiety [184]. Recently, oral administration of acetate in growing pigs had the greatest effects on appetite suppression via enhanced leptin, PYY and GLP-1 secretions [168]. Although current available evidence to support SCFA role in appetite regulation are mainly from animal models such as rats, targeting these mechanisms may be a potential strategy to improve appetite regulation through dietary intervention [186].

Findings on satiety-related effects of RS or SCFA in pigs are rather limited and inconsistent. Rapidly digestible starch stimulated insulin release that consequently inhibited feed intake and increased adiposity [187]. In contrast, slowly digestible starch facilitated protein accretion resulting in leaner and faster growing pigs [187]. Growth performance and feed intake, were reduced in weaned pigs fed slowly digestible starch containing 63% amylose [51] or a diet with 20% RS [188]. In contrast, results from another study showed that 7% of RPS reduced post weaning diarrhea in weaned piglets without affecting growth performance [15]. In other studies, growth performance of weaned [167] and finishing pigs [189] was unaffected by dietary inclusion of 30% amylose, indicating that such variations may exist due to the different RS sources and amounts consumed [190]. Incremental intake of 40% rapidly digestible starch increased fat deposition but did not affect feed efficiency when compared with feeding 40% slowly digestible starch [166]. In a study with pigs having *ad-libitum* access to feed, 30% greater RS intake did not affect feed intake pattern and growth rate of pigs likely because microbial-produced SCFA were absorbed and available as energy source thereby sparing glucose from oxidation [191]. In grower pigs, feeding diets containing 34% RS showed induced behavioral signs of increased satiety despite lack of increased plasma GLP-1 and PYY concentrations. The satiety effects observed were likely because of increased SCFA production and decreased postprandial glucose concentration and thus insulin response [192]. Separately, increased PYY secretion in response to RS supplementation is not caused by increased SCFA but rather by increased flow of intestinal digesta and neural reflexes [193]. Overall, results of the reviewed experiments indicate that dietary inclusion of up to 30% RS did not affect growth performance in growing or finishing pigs. However, dietary inclusion of RS above 30% may reduce feed intake in growing pigs. In addition, the mixed results observed for weaned pigs indicate that the overall outcome of dietary RS inclusion may also depend on starch source or the age of pigs. Combined, dietary inclusion of RS may regulate satiety and reduce feed intake in pigs without affecting growth performance, thus, improving feed efficiency.

## Conclusions

Resistant starch is the proportion of starch that escapes small intestine digestion producing mostly glucose but instead undergoes microbial fermentation in the distal small intestine and colon producing SCFA. Results of *in* *vitro* and *in* *vivo* studies support the view that RS acts as prebiotic to modulate gut microbiota by changing the intestinal microbial composition and function. In addition, specific bacteria phyla are associated with fermentation of RS and increased SCFA production particularly butyrate. Results of experiments with humans, rats, and pigs demonstrated that benefits of dietary RS on gut health may include increased markers of mucosal barrier function, immune tolerance, and increased abundance of beneficial gut bacteria. This area of research has progressed considerably; however, its benefits during disease challenge remain unclear. Indeed, additional in vivo experiments are required to provide stronger evidence linking prebiotic effects of RS with reduced PWD and mortality. Despite the potential benefits of RS fermentation in the hindgut, utilization of SCFA resulting from RS fermentation reduces energetic efficiency compared with mostly glucose from digested starch and may thus reduce growth. To achieve a balance between increased prebiotic activity and sustained growth performance of pigs, the optimum dietary inclusion of RS, most appropriate source(s) of RS, and duration of supplementation of RS need to be determined. Nevertheless, the current state of knowledge indicates that RS as a prebiotic can enhance gut health of weaned pigs, thereby reducing the incidence of PWD. The evidence linking certain bacterial taxa and its functionality to growth and feed efficiency indicates that manipulation of gut microbiota with dietary inclusion of RS may affect pig growth performance. However, reduced PWD and especially reduced mortality following such diarrhea due to dietary RS as prebiotic is more relevant economically than dietary inclusion of RS to improve feed efficiency. Effects of feeding RS on pig performance may differ depending on the health status of pigs (healthy vs. disease challenged) and gut development, thus, different optimum levels may have to be established for each growth phase and physiological stage (e.g., growth, gestation, lactation).

## Data Availability

Not applicable.

## Abbreviations

BCL6: B-cell lymphoma 6
C1S: Complement component 1S
CD4: Cluster of differentiation 4
CR2: Complement receptor type 2
CTSB: Cathepsin B
DEF1B: Defensin beta 1
GLP-1: Glucagon-like peptide 1
ICOS: Inducible T cell co-stimulator
IL: Interleukin
ITGB3: Integrin subunit beta 3
MCP2: Monocyte chemoattractant protein 2
MCT1: Monocarboxylate transporter 1
MUC: Mucin
NFKB: Nuclear factor kappa B
PolIII: RNA polymerase III
PPARγ: Peroxisome proliferator-activated receptor
PWD: Post-weaning diarrhea
PYY: Peptide YY
RPS: Raw potato starch
RS: Resistant starch
SCFA: Short-chain fatty acid
SERPING1: Serpin Family G Member 1
SREBP-1C: Sterol regulatory element binding protein 1c
TLR: Toll like receptor
TNF-α: Tumor necrosis factor alpha
